# Corrective surgery for Parkinson's disease (PD) deformity following Deep Brain Stimulation (DBS) - Is DBS a last resort?

**DOI:** 10.1186/1748-7161-10-S1-O55

**Published:** 2015-01-19

**Authors:** Jun Mizutani, Muneyoshi Fukuoka, Nobuyuki Suzuki, Seiji Otsuka, Atsushi Umemura, Yuichi Oka, Kazuo Yamada, Takanobu Otsuka

**Affiliations:** 1 Department of Orthopaedic Surgery, Nagoya City University, Japan; 2 Department of Neurosurgery, Nagoya City University, Japan

## Objective

Spinal deformity related to Parkinson's disease (PD) is severe. Due to reported high complication rate, some authors reported that PD deformity with no neurological symptoms should not undergo surgery and also reported deep brain stimulation (DBS) should be considered instead of corrective surgery for their postural disorders. However, almost of previous reports did not consider spino-pelvic parameters. If spino-pelvic harmonization is achieved even in PD deformity surgery, there is a possibility of obtaining much better surgical results. We analyzed the surgical results of PD deformity surgery focused in spino-pelvic parameters and we here report minimum two years follow up of PD deformity surgery.

## Patients and methods

Six patients with Parkinson's deformity following DBS underwent corrective surgery were enrolled this study. Mean age at surgery was 67.8 y.o. and there were 3 men and 3 women. Pelvic incidence, Pelvic tilt, SVA, lumbar lordosis, thoracic lordosis, JOA score were analyzed pre-, and post-surgery.

## Results

Five patients whose SVA was over 100 mm underwent thoracic to sacro-iliac fixation. Remaining one patient whose SVA less than 100 mm underwent L2-5 PLIF only. Mean SVA in long fixed patients was improved less than 100 mm. Lumbar lordosis in long fusion patient was improved to 30-50 degrees from flatback.

Short fusion patient's alignment following surgery was maintained. SVA was 55 mm and lumbar lordosis was 47 degrees.

## Conclusion

Although many literature have reported early breakage or complications may occur in spinal surgery in Parkinson's patients, when the PD deformity surgery is planned, considering into spino-pelvic harmonization, PD deformity spine surgery might be reduced their symptoms and the alignment might be maintained well in spite of the fusion level.

